# Ciprofloxacin in Patients Undergoing Extracorporeal Membrane Oxygenation (ECMO): A Population Pharmacokinetic Study

**DOI:** 10.3390/pharmaceutics14050965

**Published:** 2022-04-29

**Authors:** Dzenefa Alihodzic, Sebastian G. Wicha, Otto R. Frey, Christina König, Michael Baehr, Dominik Jarczak, Stefan Kluge, Claudia Langebrake

**Affiliations:** 1 Hospital Pharmacy, University Medical Center Hamburg-Eppendorf, Martinistraße 52, 20246 Hamburg, Germany; d.alihodzic@uke.de (D.A.); ch.koenig@uke.de (C.K.); baehr@uke.de (M.B.); c.langebrake@uke.de (C.L.); 2 Department of Clinical Pharmacy, Institute of Pharmacy, University of Hamburg, Bundesstraße 45, 20146 Hamburg, Germany; 3 Hospital Pharmacy, General Hospital of Heidenheim, Schloßhaustraße 100, 89522 Heidenheim, Germany; otto.frey@kliniken-heidenheim.de; 4 Department of Intensive Care, University Medical Center Hamburg-Eppendorf, Martinistraße 52, 20246 Hamburg, Germany; d.jarczak@uke.de (D.J.); s.kluge@uke.de (S.K.); 5 Department of Stem Cell Transplantation, University Medical Center Hamburg-Eppendorf, Martinistraße 52, 20246 Hamburg, Germany

**Keywords:** ciprofloxacin, population pharmacokinetics, ECMO, ARDS, NONMEM, therapeutic drug monitoring, critically ill

## Abstract

Extracorporeal membrane oxygenation (ECMO) is utilized to temporarily sustain respiratory and/or cardiac function in critically ill patients. Ciprofloxacin is used to treat nosocomial infections, but data describing the effect of ECMO on its pharmacokinetics is lacking. Therefore, a prospective, observational trial including critically ill adults (*n* = 17), treated with ciprofloxacin (400 mg 8–12 hourly) during ECMO, was performed. Serial blood samples were collected to determine ciprofloxacin concentrations to assess their pharmacokinetics. The pharmacometric modeling was performed (NONMEM^®^) and utilized for simulations to evaluate the probability of target attainment (PTA) to achieve an AUC_0–24_/MIC of 125 mg·h/L for ciprofloxacin. A two-compartment model most adequately described the concentration-time data of ciprofloxacin. Significant covariates on ciprofloxacin clearance (CL) were plasma bicarbonate and the estimated glomerular filtration rate (eGFR). For pathogens with an MIC of ≤0.25 mg/L, a PTA of ≥90% was attained. However, for pathogens with an MIC of ≥0.5 mg/L, plasma bicarbonate ≥ 22 mmol/L or eGFR ≥ 10 mL/min PTA decreased below 90%, steadily declining to 7.3% (plasma bicarbonate 39 mmol/L) and 21.4% (eGFR 150 mL/min), respectively. To reach PTAs of ≥90% for pathogens with MICs ≥ 0.5 mg/L, optimized dosing regimens may be required.

## 1. Introduction

Extracorporeal membrane oxygenation (ECMO) is a temporary life-support procedure, which ensures sufficient oxygen supply in critically ill patients when conventional methods have failed and progressive hemodynamic instability has developed. For ECMO treatment, it is important to note that the patient’s outcome is highly dependent on the management of the underlying disease. One of the main remaining challenges is the development of nosocomial infections under ECMO, which affects up to 64% of patients and is associated with a longer stay in the intensive care unit (ICU), as well as high mortality [1,2,3]. Not only the choice of the most suitable drug but also the optimal dosage of, e.g., antibiotics is of the highest relevance to improve the chances of survival of these critically ill patients and to minimize antibiotic resistance [4,5].

Ciprofloxacin is a second-generation fluoroquinolone antibiotic and is approved for the therapy of mild-to-moderate urinary and respiratory tract infections. In ICU patients receiving ECMO, ciprofloxacin (alone or in combination) is commonly used for the treatment of nosocomial pneumonia and/or severe infections [6]. It has a concentration-dependent bactericidal effect against a wide range of Gram-negative and some Gram-positive microorganisms [7]. The pharmacokinetic/pharmacodynamic (PK/PD) index that best describes the ciprofloxacin bactericidal activity is the ratio of the area under the concentration-time curve from 0 to 24 h (AUC_0–24_) to the minimal inhibitory concentration (MIC) of the causative pathogen (AUC_0–24_/MIC). Studies have suggested that a ratio of AUC_0–24_/MIC ≥ 125 mg·h/L is associated with maximal clinical success against Gram-negative infections [8,9,10]. Ciprofloxacin has low protein binding (~20–40%) and is primarily eliminated by renal excretion via glomerulus filtration and active tubular secretion processes. Non-renal clearance accounts for about one-third of elimination and includes hepatic metabolism, biliary excretion, and transmembrane secretion across the enteric mucosa [11,12].

In general, ex-vivo studies that utilized adult ECMO systems have demonstrated substantial antibiotic drug sequestration in the circuit depending on the physicochemical properties of individual drugs [13]. Shekar et al. highlighted in their review that PK changes, which are commonly associated with ECMO, result in an increased volume of distribution (Vd) and decreased drug clearance (CL) [14]. In the case of ciprofloxacin, absorption under ECMO was found to be minimal in ex-vivo or animal studies with no substantial degradation or sequestration [15,16]. Although it has been suggested that patients undergoing ECMO should receive the same dose as recommended for critically ill patients not receiving ECMO, PK studies including adult patients receiving ciprofloxacin on ECMO are still lacking.

The overall aim of this study was to gain insights into the PK of ciprofloxacin in critically ill adults receiving ECMO treatment. In particular, this entailed developing a population pharmacokinetic (popPK) model to determine accurate population pharmacokinetic parameters for ciprofloxacin, and to investigate the inter-individual PK variability and the influence of patient characteristics. Furthermore, the probability of target attainment (PTA) using dosing simulations in ICU patients was performed to develop recommendations for improved ciprofloxacin dosing regimens under ECMO.

## 2. Materials and Methods

### 2.1. Setting and Study Population

The open-labeled monocentric, prospective, observational study was approved by the local Ethics Committee of the Hamburg Chamber of Physicians (approval reference number: PV5223) and registered at the German Clinical Trials Register (DRKS00010765). Patients were recruited between July 2016 and February 2018 in the Department of Intensive Care Medicine at the University Medical Center Hamburg-Eppendorf in Germany.

Patients of both sexes were eligible for enrollment if they were ≥18 years of age, undergoing VA- or VV-ECMO, and concomitantly receiving ciprofloxacin. Written informed consent to participate in the study was obtained from each patient or their legally authorized representatives.

### 2.2. Extracorporeal Membrane Oxygenation Circuit

The ECMO system (Maquet Cardiopulmonary AG, Hirrlingen, Germany) was comprised of a Rotaflow centrifugal blood pump, a polymethylpentene (PMP) QUADROX PLS diffusion membrane hollow-fiber oxygenator, a heat exchanger, and polyvinyl chloride (PVC) tubing. All components of the circuit were albumin-heparin coated (Bioline^®^ coating, Maquet Cardiopulmonary AG, Hirrlingen, Germany). The ECMO circuit was primed with 1000 mL of Jonosteril^®^ Infusion Solution (Fresenius Kabi AG, Bad Homburg, Germany), which is a fluid- and electrolyte replacement for a balanced acid-base household. Blood pumps can generate up to 10 L/min depending on cannula size. ECMO equipment was implanted using percutaneous femoral peripheral cannulation. The blood flow and gas flow, as well as the revolutions per minutes (rpm) and the duration of ECMO, were recorded.

### 2.3. Dosing, Administration, and Data Collection

Ciprofloxacin was administered as an intravenous 30 min infusion at a dose of 400 mg every 8 or 12 h. The clinical decision to initiate ciprofloxacin treatment and a dosing schedule was undertaken by the responsible clinician.

Data related to patient demographics, renal and hepatic function, details of ECMO and renal replacement therapy (RRT) as well as laboratory variables, such as C-reactive protein (CRP), plasma bicarbonate or pH value of the blood, were collected from the electronic patient records.

The estimated glomerular filtration rate (eGFR) was calculated with various equations using the measured creatinine value and was tested as a potential covariate on ciprofloxacin PK: the Modification of Diet in Renal Disease formula (MDRD) [17], the Cockcroft–Gault formula [18], and the CKD-EPI equation (Chronic Kidney Disease Epidemiology Collaboration) [19]; on the one hand, it was calculated with the actual body surface area of the patient, and on the other hand, with a body surface area normalized to 1.73 m².

Moreover, the Sequential Organ Failure Assessment (SOFA) score, the Simplified Acute Physiology (SAPS II) score, and the Acute Physiology And Chronic Health disease classification system (APACHE II) on ICU admission were determined to describe the severity of illness of the study population and to predict the outcome of critical ill patients in the ICU.

Discrete variables were expressed as counts (percentage) and continuous variables as the median (25th and 75th percentiles), including the range of the values using Microsoft Excel.

### 2.4. Sample Collection and Measurements

For the PK study, three blood samples were collected on each of the three days during ECMO therapy within a period of seven days. Deviations occurred when (i) ciprofloxacin was discontinued or if the patient died during this period, resulting in a sample number less than nine, and (ii) if the patient subsequently received other antiinfectives in comedication to the ciprofloxacin covered in the ethical approval, resulting in the possibility of determining both antiinfectives in the same blood sample. The samples were taken separately from the infusion site as peak (1 h post-infusion), mid-dose (4 h post-infusion), and trough values (end of dose interval).

The blood samples were centrifuged (3000 rpm, 10 min), and then supernatant plasma was separated into aliquots, which were kept at −80 °C until analysis.

Ciprofloxacin plasma drug concentrations were quantified by validated high-performance liquid chromatography (HPLC), as described in Appendix A.

### 2.5. Population Pharmacokinetic Modeling

Population pharmacokinetic modeling was performed using non-linear mixed effects modeling with the software NONMEM™ version 7.4.1 (Icon Development Solution, Ellicott City, MD, USA). The first-order conditional estimation with interaction (FOCE-I) between inter-individual and residual random effects was used for parameter estimation and model development. The run manager Pirana version 2.9.7 (Certara, NJ, USA) [20] and R version 3.5.3 (R Foundation for Statistical Computing, Vienna, Austria) [21] were used for the exploratory analysis and postprocessing of the NONMEM™ output.

The likelihood ratio test (alpha = 0.05, df = 1), resulting in a difference of the objective function (dOFV) of 3.84, was used to compare nested models. The Akaike Information Criterion (AIC) was used to compare non-nested models, for which a lower score indicates a superior model [22].

The base model building started with the evaluation of one- or two-compartment pharmacokinetic models. To describe the variability among patients, inter-individual variability (IIV) terms were evaluated, assuming log-normally distributed individual PK parameters.

Since the study included samples from multiple days, inter-occasion variability (IOV) was tested on both clearance and volume parameters.

Additive, proportional, and combined residual error models were evaluated to describe the residual variability of the observed concentration-time data around the individual model predictions.

Furthermore, the shrinkage was calculated for the variability parameters, to provide information on how many patients contribute to the estimated variability parameter. A shrinkage below 20–30% was considered acceptable [23].

Demographic and clinical characteristics, which were considered physiologically plausible for affecting ciprofloxacin PK, were tested for inclusion as covariates. Therefore, a stepwise covariate modeling approach (forward inclusion–backward elimination) was chosen. Covariates (e.g., age, sex, body weight, height, BMI, body surface area, markers of liver function, serum creatinine concentration, eGFR, usage of renal replacement therapy, ECMO-related and acid-base balance parameters) were introduced into the population model using linear or power functions, with the aim of potentially explaining parts of the observed IIV among patients.

Allometric scaling models using total body weight with fixed estimated scaling parameters (coefficients of 1 for V_1_ and V_2_, 0.75 for CL and Q) were also evaluated [24].

After the inclusion of all significant covariates (alpha ≤ 0.05), a backward elimination step was used to eliminate covariates that were not highly significant (alpha ≤ 0.01, corresponding to a change in the objective function of at least 6.64) in the final model.

To assess the suitability of a model to describe the data, graphical criteria were additionally consulted. These included goodness of fit plots (GOF) of population and individual prediction (PRED and IPRED, respectively) versus observed concentrations (DV), weighted residual plots (WRES) versus time and concentration, and the prediction-corrected visual predictive check (pc-VPC) [25].

To evaluate the stability and performance of the final model, the log-likelihood profiling-based sampling importance resampling (LLP-SIR) technique was performed [26], which characterizes the estimation uncertainty and determines the 95% confidence intervals (CI) for the popPK parameters. The 2.5th and 97.5th percentiles of parameter distributions (95% CI) were compared to estimates provided by the original data set.

### 2.6. Probability of Target Attainment (PTA) Analysis

The results of the final popPK model were utilized for simulations to evaluate the PTA and to explore the alternative dosage regimen. Therefore, virtual clinical trials including 1000 patients were simulated to investigate different dosing scenarios and covariate characteristics. For each scenario, the PTA was calculated, which represented the percentage of patients achieving the AUC_0–24_/MIC target of 125 mg·h/L. During each simulation, a set of PK parameter estimates was generated, which was based on the mean and variance of the popPK parameters. First, the impact of significant covariates on the PTA, along with their combination, was assessed by calculating the PTA of the lowest and highest values observed in the study population for the MIC values of 0.06 till 2 mg/L. According to the data from the European Committee on Antimicrobial Susceptibility Testing (EUCAST) [27], the clinical breakpoint of 0.5 mg/L was used. Second, simulations for three different dosing regimens (400 mg, 600 mg, and 800 mg every 8 h, respectively) were tested to derive dose recommendations for critically ill adult ECMO patients. A PTA of at least 90% was considered appropriate.

## 3. Results

### 3.1. Study Population and Pharmacokinetic Data

Seventeen patients (1 female and 16 males) were included in the study. The population study group revealed normal values for age, weight, and height. The patients were severely ill (median SAPS II = 48, 15–73). Five patients (29%) received 400 mg every 12 h, and twelve patients (71%) were treated with 400 mg every 8 h. For the PK study, 147 plasma samples were measured, and an average of eight samples (range: 3–17) per patient was available. One sample was excluded due to uncertainty regarding the exact sampling time, and seven data points were considered erroneous due to implausibly high or low through or peak levels. Thus, the final data set consisted of 139 plasma samples. Patient and ECMO characteristics are displayed in Table 1.

### 3.2. Population Pharmacokinetic Analysis

A two-compartment model with first-order elimination most adequately described the concentration-time data of ciprofloxacin in adult ECMO patients. It was superior compared to the one-compartment model (AIC: −11.099). The residual variability was described by a proportional error model. Inter-individual variability (IIV) was included on clearance (CL), central volume of distribution (V_1_), and intercompartmental clearance (Q), and it considerably improved the model. The IIV on the peripheral volume of distribution (V_2_) tended to zero and thus was not included in the model. The inclusion of an inter-occasion variability (IOV) on CL markedly improved the model (dOFV = −19.512), corresponding to a noticeable reduction of the proportional error from 48.6% to 12.2%. Here, each administration was defined as a new occasion.

The first included covariate that considerably improved the base model statistically as well as graphically was the plasma bicarbonate value on ciprofloxacin clearance, which reduced the OFV by 22.5 (*p* = 2 × 10^−^^6^) and the IIV on CL from 46.6% to 29.3%. A further reduction by 4.468 points (*p* = 0.035) in the OFV was obtained by including the eGFR on CL, calculated with the CKD-EPI equation using the actual body surface. After the backward elimination, CKD-EPI was retained in the model because it is biologically plausible. It reduced the IIV of CL further to 25.8%, and the improvement in the graphical criteria was noted. CKD-EPI was the superior covariate compared to the Cockcroft-Gault or MDRD formula.

Allometric scaling of the PK parameters by body weight did not improve the model substantially. Thus, it was not included in the final model.

None of the other 55 tested covariates, especially ECMO-related ones (e.g., ECMO cannulation, blood/gas flow, and revolutions per minutes or the duration of ECMO), could be identified as significant.

The final covariate relationship on clearance is represented by:

CL = CL_typ_·(1 + 0.0571·(BICARB − 25.7 mmol/L))·(1 + 0.0038·(eGFR − 40.83 mL/min)) (1)

where CL_typ_ is the typical value of clearance, 0.0571 describes the effect of the plasma bicarbonate (BICARB) of the patient on clearance, and 0.0038 describes the effect of the estimated glomerular filtration rate (eGFR) calculated by the CKD-EPI equation on clearance.

For the final model, the typical CL for a patient with plasma bicarbonate of 25.7 mmol/L and an eGFR of 40.83 mL/min was estimated to be 12.9 L/h (IIV: 25.8% CV). The central and peripheral volume of distribution (V_1_, V_2_) were 73.4 L (IIV: 53.9% CV) and 87.6 L, respectively, while the intercompartmental clearance (Q) was estimated to be 38.8 L/h (IIV: 34.1% CV). Individual clearance and total volume of distribution (V_1_ + V_2_) ranged from 3.7 to 36.3 L/h and 117.2 to 228.9 L, respectively, in the studied population.

The parameter estimates of the final model are presented in Table 2, together with the results from the LLP-SIR. The final model estimates were in all cases within the 95% CI.

### 3.3. Model Evaluation

**Figure 1:**
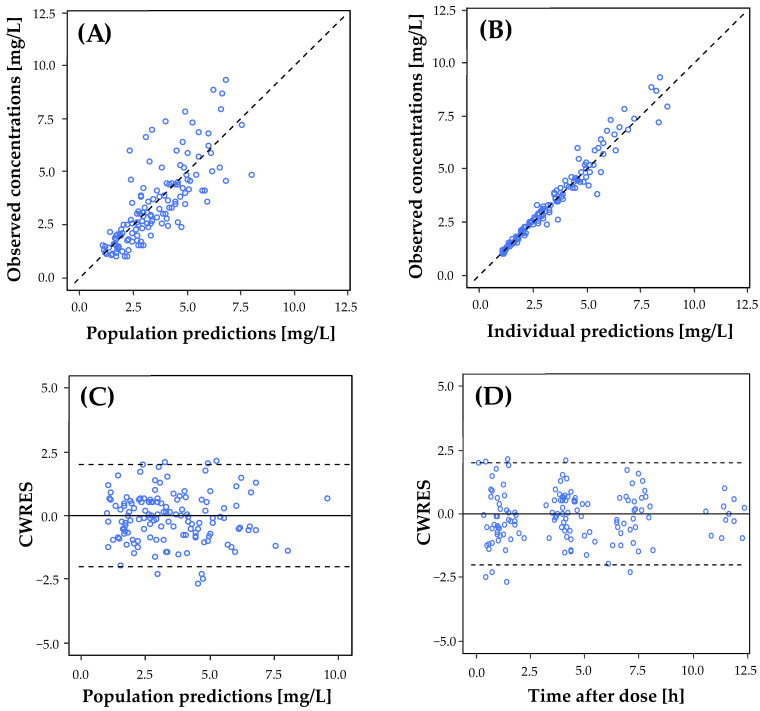
The goodness-of-fit (GOF) plots of the final PK model for ciprofloxacin in adult ECMO patients. The observed ciprofloxacin concentration versus (**A**) the population-predicted concentrations, and (**B**) the individual-predicted concentrations; dashed line in (**A**,**B**): line of identity. The conditional weighted residuals (CWRES) versus population predicted concentration (**C**), and time after the first dose (**D**). Blue circles represent individual data points; solid black line in (**C**,**D**): conditional weighted residuals are equal to 0; dashed black lines in (**C**,**D**) represent 95% confidence interval.

The goodness-of-fit plots for the final model are displayed in Figure 1 and depicted that the population predictions (A) and individual predictions (B) of the final model were evenly distributed around the identity line when plotted against the observations. Indeed, the scatter plots of the conditional weighted residuals (CWRES) were normally distributed over the *x*-axis when plotted against the population predicted concentration (C) and the time after dose (D). These plots revealed a good predictive performance.

**Figure 2:**
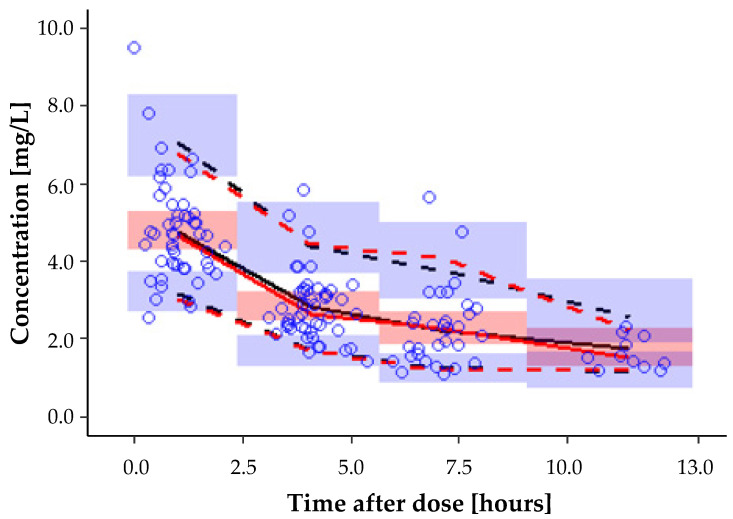
The visual predictive check (prediction-corrected) of the final PK model, showing the median, 5th, and 95th percentile of observed data (red lines) and the median, 5th, and 95th percentile of predicted data (black lines). The circles represent the prediction-corrected observed concentrations, and the shaded areas represent 95% confidence intervals.

The prediction-corrected visual predictive check (pc-VPC) of the final model is presented in Figure 2 and indicates high agreement between the observed and model-predicted concentrations.

### 3.4. Probability of Target Attainment (PTA)

**Figure 3:**
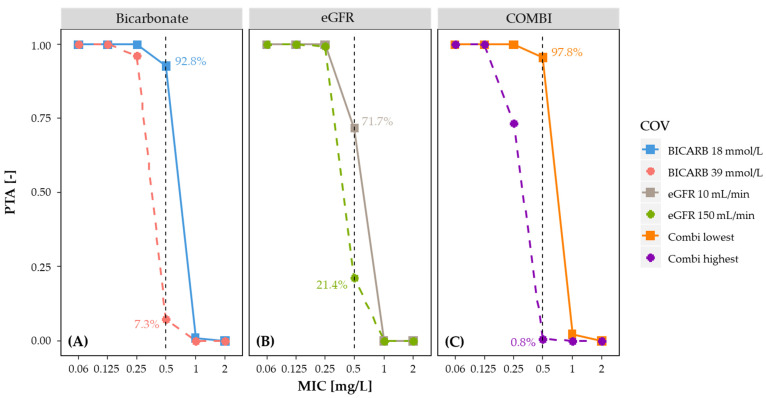
The impact of (**A**) the bicarbonate value, (**B**) the eGFR, and (**C**) the combination of the highest and lowest value of these two covariates (in the study population) on the PTA. The percentages represent the respective PTA at a breakpoint of 0.5 mg/L (dotted vertical line). A PTA ≥ 90% was considered satisfactory.

As shown in Figure 3, for pathogens with an MIC of ≤0.25 mg/L, a PTA of ≥90% was attained. However, for pathogens with an MIC of ≥0.5 mg/L, plasma bicarbonate ≥ 22 mmol/L, or eGFR ≥ 10 mL/min decreased the PTA below 90%, steadily declining to a PTA of 7.3% (plasma bicarbonate 39 mmol/L) and a PTA of 21.4% (eGFR 150 mL/min), respectively. Regardless of the covariate value, at an MIC ≥ 1 mg/L the PTA was unacceptably low (<1%).

In these simulations, 92.8% of the patients with a plasma bicarbonate of 18 mmol/L and 7.3% of patients with a plasma bicarbonate of 39 mmol/L achieved the target of AUC_0–24_/MIC ≥ 125 mg·h/L when using dosing regimens of 400 mg every 8 h along with the standard EUCAST MIC susceptibility breakpoint of 0.5 mg/L (Figure 3). Similar tendencies were obtained in the case of the second covariate: 71.7% of the patients with an eGFR of 10 mL/min and 21.4% of patients with an eGFR of 150 mL/min attained the target, respectively.

The covariate effects potentiate in the presence of high plasma bicarbonate concentrations and a higher eGFR, so that even for pathogens with an MIC of 0.25 mg/L, the PTA decreased below 75%. Correspondingly, for pathogens with an MIC of 0.5 mg/L, the PTA decreased below 1%.

**Figure 4:**
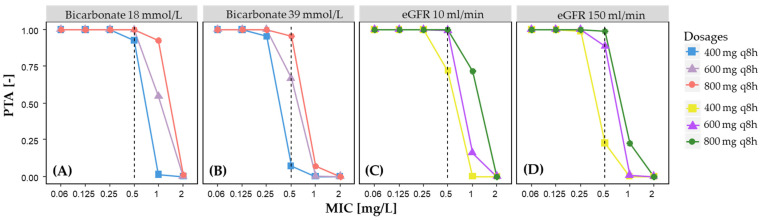
The impact of three different dosing regimens on the PTA, depending on (**A**) the lowest bicarbonate, (**B**) the highest bicarbonate, (**C**) the lowest eGFR, and (**D**) the highest eGFR values in the study population and the respective MIC value. The dashed vertical line represents the breakpoint of 0.5 mg/L. A PTA ≥ 90% was considered satisfactory.

The PTA for achieving an AUC_0–24_/MIC ratio of 125 mg·h/L for three different ciprofloxacin doses depending on the covariate value is described in Figure 4. At an MIC of 0.5 mg/L, increasing the dose from 400 mg to 600 mg every 8 h will increase the PTA from 7.3% to 66.7% in patients with a plasma bicarbonate value of 39 mmol/L. With a further increase to 800 mg every 8 h, the PTA achieves a value of 95.4%. Similar results can also be observed in other scenarios, which are displayed in Figure 4 and indicate that an elevated dosage is required depending on the plasma bicarbonate and the eGFR value, in addition to the target MIC value of the pathogen.

## 4. Discussion

To the best of our knowledge, this is the first study to describe the PK of ciprofloxacin of critically ill adult ECMO patients using a popPK approach. Our results indicate that a two-compartment model with first-order elimination was optimal to describe the concentration-time data of ciprofloxacin in ECMO patients. Hence, it agrees with previous observations in other ICU population groups without using ECMO [28,29,30,31,32]. In contrast to previous ciprofloxacin studies not on ECMO, this was the first time plasma bicarbonate was introduced as a covariate in the final model.

We found the total Vd (V_1_ + V_2_) of 161 L to be slightly higher in comparison to 106 L, and the CL of 12.9 L/h to be slightly lower than the 19 L/h reported by Khachmann et al. [29], who examined the PK of ciprofloxacin in ICU patients not on ECMO and whose ciprofloxacin model is most similar to ours. Similar findings were obtained by comparing our results to the observations of three other studies [28,31,32], who also observed ICU patients on similar dosages not on ECMO. Our reported Vd was higher than their total range of 62.0–116.7 L. Further, the CL reported in our study was lower than the CL of 13.6–42.0 L/h as the total range of the other studies.

When compared to healthy volunteers [33], the typical value for CL of our study population was lower by half, which might have been the result of renal and/or hepatic dysfunction in critically ill patients.

Consequently, our study confirmed the hypothesis from Shekar et al. [14], who reported an increased Vd and decreased CL for lipophilic antimicrobials in ECMO patients. This can be explained, on the one hand, by the larger surface area and drug sequestration in ECMO circuits and, on the other hand, by the severe critically illness and organ dysfunction of our patients. The sequestration of drugs occurs particularly for lipophilic and high protein-bounded drugs [13,15]. Despite the lipophilic properties of ciprofloxacin (log P 2.3), an ex-vivo study [15], along with an in-vivo ovine model [16], observed only a minimal loss of ciprofloxacin from the ECMO circuit and therefore suggested only a low risk of sequestration. The reason might be the low to moderate protein binding varying from 20–40%. Thus, the authors revealed that the extent of protein binding may determine circuit drug loss for drugs with similar lipophilicity. However, even if the used ECMO system is comparable to the one we used, it is difficult to transfer PK data from ex-vivo or animal studies to critically ill adults. Hence, due to the larger surface area in addition to its lipophilic characteristics, this deposition could have resulted in a critical-illness-related increase in the Vd. The presence of organ failure in more than half of the patients (10 patients with renal insufficiency and 11 patients with hepatic failure) contributed to the reduced total CL, which we observed in our study population. To optimize fluid balance and support renal function, eight patients received continuous veno-venous hemofiltration (CVVH) in addition to the ECMO circuit. However, the magnitude of the difference in PK parameters was relatively small, thus suggesting that the observed difference may not be clinically relevant.

As seen in our results, we observed a high inter-individual variability (IIV), which can be partially explained by the inclusion of two significant covariates on CL in the final model. As the most significant covariate, plasma bicarbonate was identified and revealed, with high values, an increased CL and decreased AUC_0–24_ of ciprofloxacin. Bicarbonate is an electrolyte and a negatively charged ion that is used by the body to help maintain the body`s acid-base (pH) balance. To increase the pH, the renal system reabsorbs bicarbonate and secretes acids into the urine. At a physiologic pH of 7.4, ciprofloxacin exists primarily as zwitterion and is practically uncharged. It has an amino group (piperazine) with a pK_a_ of 8.74 and a carboxylic acid group with a pK_a_ of 6.09 [34]. Depending on the pH value, ciprofloxacin is ionized and tubularly secreted via organic anion and cation transport systems [35], which conversely means that a plasma bicarbonate value beyond the physiological range increases the clearance. This finding suggests that in states of alkalosis or acidosis, the clearance increases, which is associated with the risk of underexposure and eventually treatment failure. However, our model only described the unilateral effect in cases of alkalosis, and therefore the extent of increased drug elimination in the case of acidosis needs to be further investigated.

Additionally, another part of the remaining variability on CL can be explained by the effect of eGFR calculated with the CKD-EPI equation. Since ciprofloxacin is mainly eliminated by the renal route, it was expected to have an influence on the clearance and dosing of ciprofloxacin. This finding is consistent with previously reported ciprofloxacin popPK models [29,31,36] in other populations in which a measure for renal function was also found to be a relevant covariate. Consequently, plasma bicarbonate potentially reflects the pH-dependent tubular secretion of ciprofloxacin in our final model, whereas eGFR reflects the impact of glomerular filtration on CL. Both covariates were positively correlated with ciprofloxacin CL, whereas ECMO-related parameters were not significantly associated with PK parameters. Therefore, our study suggests that ECMO does not have a major effect on ciprofloxacin PK in adult ECMO patients. Considering the reported PK/PD target for AUC_0–24_/MIC of 125 mg·h/L [8,9,10], the high-exposure dosing of ciprofloxacin in adult ICU patients (1200 mg/day) would be considered appropriate for pathogens with an MIC value equal or below to 0.25 mg/L. At the clinical breakpoint of 0.5 mg/L, our simulations demonstrated that only 7.3% (for a plasma bicarbonate of 39 mmol/L) and 21.4% (for an eGFR of 150 mL/min) of all simulated patients achieved the PTA target. For pathogens with an MIC > 0.5 mg/L, ciprofloxacin is not a therapeutic option. This is in line with previous observations with levofloxacin [37].

In relation to our patient cohort, one patient with a plasma bicarbonate value above 34 mmol/L and an eGFR above 142 mL/min, and another patient with the combination of a plasma bicarbonate value below 20 mmol/L and an eGFR below 30 mL/min, were observed. A total of six patients (35.3%) were able to achieve the target AUC_0–24_/MIC value ≥ 125 mg·h/L, assuming an MIC of 0.5 mg/L. Correspondingly, eleven patients (64.7%) had target values below 125 mg·h/L, with two of these patients (18.2%) with values below 100 mg·h/L.

To reach the recommended AUC_0–24_/MIC ratio in these patients, higher doses of ciprofloxacin up to 800 mg every 8 h would be necessary but are outside the scope of approval. Of course, higher doses may increase potential dose-dependent toxicities of ciprofloxacin [38]. Therefore, such a procedure cannot be generally recommended but could be accompanied by appropriate close-meshed therapeutic drug monitoring (TDM) if there are no therapeutic alternatives.

Nevertheless, some limitations of this study shall be discussed.

First, this study was limited by its small sample size (*n* = 17), so the conclusions that can be drawn are limited to a very narrow patient cohort. However, Shekar et al. [39] proposed that a minimum of 12 patients would be sufficient for a popPK analysis in ECMO patients. Second, it was not possible to measure ciprofloxacin concentrations pre and post oxygenator, which were initially planned to better characterize potential drug loss in the ECMO circuit, for technical and clinical reasons. Additionally, only the total fraction of ciprofloxacin was measured because ciprofloxacin has a low protein binding of 20–30%. Third, the infusion times were not precisely documented, which led to a higher residual error. Therefore, we revealed in our previous work [40] that erroneous records, even with little uncertainty in documented time, considerably impact the accuracy and the precision of estimated PK parameters on the population and individual level. The correct documentation is vital and should deserve higher attention, even in challenging study settings such as the ICU. Fourth, the results obtained for the PK parameters were compared to previous publications on ICU patients because there was no direct control group in our study population. Fifth, no MIC data were measured in our population so that the general information from EUCAST was used. Lastly, the dosing recommendations of >1200 mg/day at a breakpoint of 0.5 mg/L are only based on simulations and have not been externally validated.

## 5. Conclusions

To the best of our knowledge, our work offers the first insights into the pharmacokinetics of ciprofloxacin in critically ill adults under ECMO treatment. The typical PK parameters only slightly differed compared to other critically ill patients, suggesting that the observed differences may not be clinically significant. Plasma bicarbonate was identified as the most significant covariate, potentially reflecting the pH-dependent tubular secretion of ciprofloxacin, whereas eGFR as a second covariate reflected the glomerulus filtration of ciprofloxacin. To reach PTAs of ≥90% for pathogens with ciprofloxacin MICs ≥ 0.5 mg/L, larger doses or shorter dosing intervals may be required. Given the high variability of ciprofloxacin PK under ECMO and bacterial susceptibility, therapeutic drug monitoring may help to further optimize individual ciprofloxacin dosing in clinical practice. Further investigations of antibiotics undergoing tubular secretion are warranted in ECMO patients.

## Figures and Tables

**Table 1:** The demographic and clinical data.

Variable	Median (IQR)	Range
Age (years)	57 (51–63)	25–73
Weight (kg)	90 (80–103)	66–123
Height (m)	1.8 (1.75–1.82)	1.60–1.97
BMI (kg/m²)	29.3 (26.1–30.9)	20.4–36.7
**Scores**		
SOFA score	13 (10–14)	3.0–19
SAPS II score	48 (38–52)	15–73
APACHE II score	32 (21–33)	14–46
**Blood chemistry, serum levels**		
Serum creatinine (mg/dL)	1.3 (0.88–2.0)	0.25–4.10
GFR (CKD-EPI) (ml/min)	46.8 (25.4–79.6)	10.7–143
Serum albumin concentration (g/L)	21.3 (16.2–24.9)	10.4–31.0
Total bilirubin (mg/dL)	1.0 (0.7–2.0)	0.2–24.4
AST level (U/L)	56 (39.5–134)	21–1568
ALT level (U/L)	56 (32–118.5)	11–543
C-reactive protein (mg/L)	128 (73.5–202.3)	8–475
Bicarbonate (mmol/L)	26.6 (24.3–29.7)	18.0–39.0
pH value	7.43 (7.39–7.46)	7.25–7.58
**ECMO parameter**		
Length of ECMO therapy (days)	13 (9–16)	(4–28)
Blood flow (L/min)	4.4 (3.9–5.2)	2.2–6.73
Gas flow (L/min)	4.25 (3.5–5.1)	0.9–10
Revolutions per minutes (RPM)	3460 (3185–3804)	2270–5000
PaO_2_/FiO_2_	102.5 (71.1–143.3)	26.8–463
**Ciprofloxacin serum concentrations (mg/L)**		
Peak	4.55 (3.69–5.9)	2.38–12.14
Mid-dose	2.57 (1.97–3.45)	1.06–9.3
Through	1.53 (1.11–2.57)	0.77–6.88
	**Number (%)**	**Range**
**Sex**		
Male	16 (94)	NA
Female	1 (6)	NA
**Indication for ECMO**		
ARDS	6 (35)	NA
Global respiratory insufficiency	1 (6)	NA
Pulmonary fibrosis	1 (6)	NA
Cardiac decompensation	1 (6)	NA
Bridge to transplant	1 (6)	NA
Cardiogenic shock (STEMI/NSTEMI)	7 (41)	NA
**ECMO type**		
Veno-arterial (VA)	8 (47)	NA
Veno-venous (VV)	8 (47)	NA
Veno-veno-arterial (VVA)	1 (6)	NA
**Use of continuous hemodiafiltration**		
	8 (47)	NA
**Outcome**		
Survived	5 (29)	NA
Deceased	12 (71)	NA

Abbreviations: BMI—body mass index, SOFA—Sequential Organ Failure Assessment, SAPS II—Simplified Acute Physiology Score, APACHE II—Acute Physiology And Chronic Health Score, GFR (CKD-EPI)—glomerular filtration rate calculated using the equation of Chronic Kidney Disease Epidemiology Collaboration, AST—aspartate aminotransferase, ALT—alanine aminotransferase. NA—not applicable, ARDS—Acute Respiratory Distress Syndrome, STEMI—ST-segment Elevation Myocardial Infarction, and NSTEMI—Non-ST-segment Elevation Myocardial Infarction.

**Table 2:** The population pharmacokinetic parameter estimates from the base and final model of ciprofloxacin, as well as the LLP-SIR results.

	Base Model		Final Model		LLP-SIR
	Estimate	Shrinkage	Estimate	Shrinkage	95% CI
	(% RSE)	(%)	(% RSE)	(%)	
**Fixed effects**					
CL (L/h)	13.6 (12)	-	12.9 (7.6)	-	11.2–14.7
V_1_ (L)	73.2 (16)	-	73.4 (15)	-	53.3–95.5
Q (L/h)	39.4 (33)	-	38.8 (30)	-	25.7–57.7
V_2_ (L)	88.3 (17)	-	87.6 (16)	-	66.1–115.1
COV_CL_BICARB (L/mmol)	-	-	0.0571 (12)	-	0.0334–0.0770
COV_CL_eGFR (min/mL)	-	-	0.0038 (29)	-	0.0003–0.0074
**First level of random effects**					
IIV CL (CV%)	46.4 (20)	0	25.8 (19)	4	18.7–36.0
IIV V_1_ (CV%)	48.1 (18)	14	53.9 (17)	12	33.1–76.1
IIV Q (CV%)	38.7 (64)	47	34.1 (76)	52	4.2–69.6
IIV V_2_ (CV%)	3.2 FIX	94	-	-	-
IOV CL (CV%)	18.5 (17)	-	17.2 (21)	-	11.3–23.3
**Second level of random effects**					
Prop. σ (CV%)	12.2 (17.3)	29	12.1 (21.9)	28	10.2–15.2
OFV	29.054	-	2.082	-	-

Abbreviations: CL—the clearance, V1—the central volume of distribution, Q—the inter-compartmental clearance, V2—the peripheral volume of distribution, COV_CL_BICARB—the typical pharmacokinetic parameter for the first covariate on the clearance (bicarbonate value), COV_CL_eGFR—the typical pharmacokinetic parameter for the second covariate on the clearance (estimated glomerular filtration rate), IIV—the inter-individual variability, IOV—the inter-occasion variability, Prop. σ^2^—the residual variability calculated as a proportional error, and CV—the variability estimates were transformed into coefficient of variation (CV) values as follows: %CV = sqrt(exp(OMEGA) − 1) × 100, OFV—the objective function value, RSE—the relative standard error, LLP-SIR—the log-likelihood profiling-based sampling importance resampling, and CI—the confidence interval.

## Data Availability

Data can be received from the corresponding author on reasonable request.

## Appendix A

HPLC analyses were carried out by using a Shimadzu chromatographic system with a Photo Diode Array Detector (PDA) and a reverse-phase column (Atlantis^®^ dc18, 3 µm, 4.6 × 150 mm) with gradient elution of the mobile phase. The mobile phase consists of 0.05% formic acid in water and 0.05% formic acid in acetonitrile at a flow rate of 1.0 mL/min. The quantification was performed with the internal standard (moxifloxacin) method. Detection was done at a wavelength of 350 nm. Plasma samples were prepared by dilution involving a protein precipitation step for plasma, and an acetonitrile-methanol mixture (50/50 *v*/*v*): 250 µL of the plasma sample was supplemented with 50 µL internal standard and 500 µL acetonitrile-methanol mixture. The solution was vortexed for 10 s and centrifugated (4000 rpm, 5 min). For HPLC analysis, 200 µL of sample was diluted with 600 µL of water.

The coefficient of variation of this method for the intra-day precision was between 0.21% and 0.7% for ciprofloxacin concentrations of 1–8 mg/L. The accuracy was >95% with a deviation < 15% for all tested concentrations. The lower limit of quantification was 0.5 mg/L, and the lower limit of detection was 0.4 mg/L.
